# A potential large and persistent black carbon forcing over Northern Pacific inferred from satellite observations

**DOI:** 10.1038/srep43429

**Published:** 2017-03-07

**Authors:** Zhongshu Li, Junfeng Liu, Denise L. Mauzerall, Xiaoyuan Li, Songmiao Fan, Larry W. Horowitz, Cenlin He, Kan Yi, Shu Tao

**Affiliations:** 1 Laboratory for Earth Surface Processes, College of Urban and Environmental Sciences, Peking University, Beijing, China; 2 Woodrow Wilson School of Public and International Affairs, Princeton University, Princeton, NJ, USA; 3 College of Environmental Science and Engineering, Peking University, Beijing, China; 4 Department of Civil and Environmental Engineering, Princeton University, Princeton, NJ, USA; 5 NOAA Geophysical Fluid Dynamics Laboratory, Princeton, New Jersey, USA; 6 Department of Atmosphere and Oceanic Sciences, University of California at Los Angeles (UCLA), Los Angeles, CA, USA

## Abstract

Black carbon (BC) aerosol strongly absorbs solar radiation, which warms climate. However, accurate estimation of BC’s climate effect is limited by the uncertainties of its spatiotemporal distribution, especially over remote oceanic areas. The HIAPER Pole-to-Pole Observation (HIPPO) program from 2009 to 2011 intercepted multiple snapshots of BC profiles over Pacific in various seasons, and revealed a 2 to 5 times overestimate of BC by current global models. In this study, we compared the measurements from aircraft campaigns and satellites, and found a robust association between BC concentrations and satellite-retrieved CO, tropospheric NO_2_, and aerosol optical depth (AOD) (R^2^ > 0.8). This establishes a basis to construct a satellite-based column BC approximation (sBC*) over remote oceans. The inferred sBC* shows that Asian outflows in spring bring much more BC aerosols to the mid-Pacific than those occurring in other seasons. In addition, inter-annual variability of sBC* is seen over the Northern Pacific, with abundances varying consistently with the springtime Pacific/North American (PNA) index. Our sBC* dataset infers a widespread overestimation of BC loadings and BC Direct Radiative Forcing by current models over North Pacific, which further suggests that large uncertainties exist on aerosol-climate interactions over other remote oceanic areas beyond Pacific.

Black carbon is a minute carbonaceous particle, originating from incomplete combustion of fossil fuels and biofuels and biomass burning. It strongly absorbs sunlight and imposes considerable positive radiative forcing on global and regional climate through multiple ways^1^. In a recent assessment, total climate forcing of BC is estimated to be 1.1 Wm^−2^ and it is the second largest climate forcer after carbon dioxide^2^, while other studies suggest the radiative forcing of BC might be lower^3^,^4^,^5^,^6^. This difference is partially caused by the uncertainties in our understanding of BC emissions, atmospheric aging and wet removal processes^2^,^7^,^8^,^9^, resulting in a large bias in BC simulation, particularly over remote areas.

Since Asia is the largest source of BC aerosols in the world^2^,^10^, high loading of BC aerosols in Asia is found responsible for a local warming trend as well as enhanced air pollution^11^,^12^. Asia is also believed to be the dominant contributor to BC burden over North Pacific. Several important meteorological conditions facilitate the export of BC aerosols out of mainland Asia, such as lofting in the Warm Conveyor Belt (WCB) of mid-latitude cyclones, transport within the boundary layer, and orographic lifting^13^,^14^. Chemical transport models are used to quantify the extent of trans-pacific influences as well as to acquire the spatiotemporal distribution of BC aerosols in Asian downwind regions. However, large spatiotemporal discrepancies have been found when comparing modeled BC concentrations to the HIAPER Pole-to-Pole Observations (HIPPO) over Pacific. This indicates unresolved uncertainties in global transport models^6^,^15^, which threatens the reliability of current climate projections on Pacific. In addition, since each aircraft campaign covers only a few days, the intercepted short-term BC concentrations may be insufficient to represent the highly variable BC fields over the entire Pacific. Therefore, acquiring more BC measurements over remote oceans can significantly expand our understanding on BC’s trans-pacific transport characteristics and the associated climate forcing.

Satellite observation has become an increasingly valuable source for atmospheric research. Gas absorbs light at fixed wavelengths due to its vibrational and/or rotational transitions. Instruments on board satellites collect atmospheric transmission information on such wavelengths. Column concentration of certain gas can be accurately retrieved based on atmospheric transmission information on specific wavelengths. However, aerosols are not singular chemicals as gases and have diverse absorption and scattering spectrums, which are subject to their chemical composition, size distribution and physical mixing states. Algorithms have been successfully developed to retrieve Aerosol Optical Depth (AOD), as an indicator for the total absorbing and scattering effects of aerosols at certain wavelengths, e.g., 550 nm AOD products provided by Moderate Resolution Imaging Spectroradiometer (MODIS)^16^. Algorithms are in progress regarding Absorption Aerosol Optical Depth (AAOD), which represents only the absorbing effects of aerosols. Black carbon, dust, and organic carbon aerosols are responsible for such absorbing effects. So far, Ozone Monitoring Instrument (OMI) provides a set of online global AAOD data but it shows poor performances when validated by ground-based Aerosol RObotic NETwork (AERONET) AAOD^17^,^18^. Nonetheless, given the exorbitant price of atmospheric measurements on a global scale, satellite observations are expected to be valuable data sources for global knowledge on atmospheric sciences, including black carbon.

Direct measurement of BC by satellite is challenging because BC aerosols do not have a distinctive absorption spectrum to be easily identified. However, during the evolution of BC aerosols in the atmosphere, there are several air pollutants sharing similar emission sources, transport features and scavenging processes as BC. For instance, incomplete combustion process produces black carbon along with carbon monoxide, whereas sulfur dioxide, nitrogen oxides and other aerosols are also co-emitted. It has been widely acknowledged that carbon monoxide and other aerosols coexist with BC aerosols in both source regions and remote regions^19^,^20^,^21^,^22^,^23^. During Asian export event, a strong correlation among PAN, C_2_H_2_, CO and SO_2_ has been found^24^ and BC aerosols travel with these air pollutants in parcels^20^,^22^. In wet deposition event, hydrophobic BC aerosols act like carbon monoxide while aged BC aerosols act like sulfate aerosols. Although there has been no affirmative study, there is likely to be an association between the concentrations of BC mass and other satellite-retrievable air pollutants. Furthermore, such a relationship, if exists, could be employed to infer BC mass from other air pollutants in places and periods of time without available BC measurements.

Hereinafter, we examine the relation between BC’s column mass concentration (derived from the HIAPER Pole-to-Pole Observation (HIPPO) and A-FORCE aircraft measurements) and potentially coexisted pollutants (CO, SO_2_, tropospheric NO_2_, aerosols and absorbing aerosols from satellite observations) as well as some meteorological factors over North Pacific. We then simplify the empirical relationship according to the robustness test of each factor and discard factors that prove insignificant. Finally, we apply the condensed relationship to infer a satellite-based column BC inference (sBC*) over North Pacific. Although aircraft measurements only present us with fragmentized spatiotemporal information on BC, they serve as a bridge between the real-time satellite observations and the actual atmospheric compositions, and the inferred dataset based on satellite observations provides unprecedented information regarding the spatiotemporal distribution of BC aerosols over Northern Pacific for a much longer time span.

## Results

### Derivation of satellite-based black carbon concentration

185 vertical profiles of BC over North Pacific were extracted from HIPPO and A-FORCE measurements and then merged into column BC concentration ready for comparison with satellite observation, which includes carbon monoxide, tropospheric nitrogen dioxide and aerosol optical depth. The empirical relationship we derived for sBC* follows:


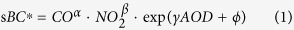


where sBC* is the predicted **s**atellite-based column **BC** approximation (unit: ng/m^2^); AOD is the average aerosol optical depth from MODIS on Aqua and Terra^16^; CO (unit: 10^18^ molecules/cm^2^) and NO_2_ (unit: 10^15^ molecules/cm^2^) are satellite retrievals from AIRS^25^ and OMI^26^ respectively. Daily satellite data with identical geological coordinates to aircraft measurements are selected. Regression reveals robust correlation (R^2^ = 0.80) between satellite observations and aircraft measurements and the best-fit parameters are: *α* = 3.35, *β* = 0.79, *γ* = 5.4, *ϕ* = 8.3 (CO < 2.3) and *α* = 0, *β* = 0.54, *γ* = 4.0, *ϕ* = 11.7 (CO > 2.3).

**Figure 1:**
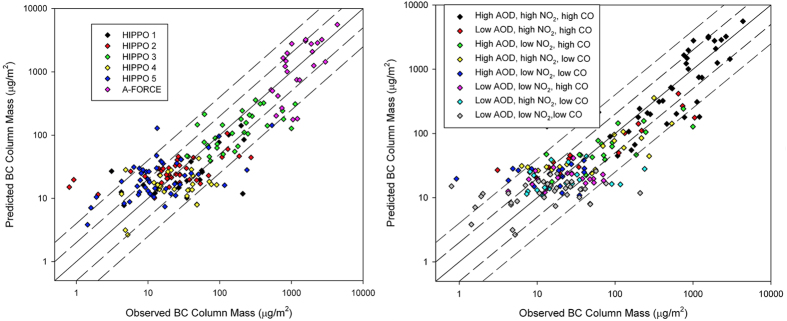
Scatter plots of measured and predicted BC column mass concentrations. (**a**) categorized by different aircraft campaigns; (**b**) categorized by relative abundances of CO, NO_2_ and AOD values.

In general, column BC mass predicted from the satellite-based model agrees well with A-FORCE and all HIPPO measurements, which suggests that the model is applicable to both Pacific Ocean and Asian outflow regions covering all seasons. As shown in Fig. 1a, a robust association (R^2^ = 0.80) is found between BC column mass from aircraft measurements and BC column mass inferred by the model (Equation (1)) using coincided satellite observations. Predicted BC (sBC*) shows extraordinary consistency with aircraft measurements in the high range (>100 μg·m^−2^), which is a good tracer for BC plumes. Figure 1b further categorizes data points into eight groups according to their relative abundance of coexisted pollutants. Each of the three satellite variables are divided into a “low” group and a “high” group by their median values and eight groups are yielded by full permutation. Observed high BC plumes are found to coincide with heavy loadings of coexisted air pollutants. Both quantitative and qualitative analysis strongly supports the assumption that BC aerosols and other air pollutants coexist in remote air, especially in plumes with high BC loadings. Such coexistence could be attributed to their similar emission source, evolution process and transport pathway. For instance, both CO and NO_2_ are generated from combustion sources, but with different indications to BC emissions. Large amount of CO indicates substantial incomplete combustion that usually co-emit BC. NO_x_ is a good indicator of anthropogenic emissions, especially for traffic emissions in which both BC and NO_x_ are released from diesel vehicles. Aerosol optical depth over the western Pacific is mainly contributed from sulfate and organic aerosols^27^. Higher levels of AOD over the Pacific show significant transport of hydrophilic aerosols, indicating the scarceness of precipitation over the source and the western Pacific^28^. Therefore, when satellites measure increased CO, NO_2_ and AOD over the Pacific, there is a high chance that BC is being exported to the Pacific Ocean.

Thereinafter, we apply the retrieving model to locate BC plumes over North Pacific and we can rely on the daily column BC dataset inferred from satellite observations to improve our understanding on the spatiotemporal distribution of BC aerosols over North Pacific. Although aircraft measurements are sparse over remote oceans, satellite retrievals are much more available and equation (1) can expand our knowledge on the abundance of black carbon aerosols in terms of both temporal and spatial resolution. The global BC dataset based on equation (1) is called sBC* as satellite BC proxy.

### Spatiotemporal distribution of black carbon over North Pacific

**Figure 2:**
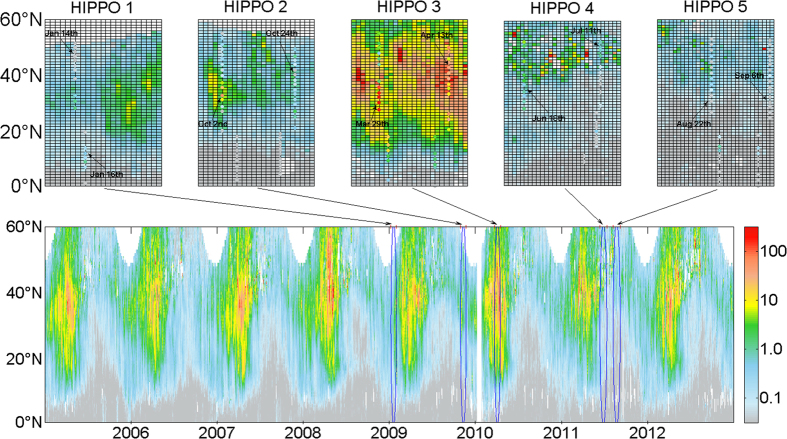
Time series of meridional sBC* distribution over middle North Pacific (160°E–160°W) average from 2005–2012 (unit: μg·m^−2^). Upper panel shows the sBC* distribution over five HIPPO campaign periods and dots indicate HIPPO measurements.

In Fig. 2, time-series of the distribution of sBC* averaged from 160°E to 160°W are shown. Generally, high sBC* events occur within 10°N to 40°N, corresponding to massive BC emissions from Southeast Asia and East Asia^10^. Plumes are occasionally observed north of 40°N, with both intensity and frequency greatly reduced, indicating the forest fire plumes transported from Siberia^29^. At lower latitudes south to 10°N, however, sBC* loadings are very low, consistent to observations sampled by multiple HIPPO missions. From a seasonal perspective, sBC* loadings are highest in spring, an order of magnitude larger than that in autumn. This pattern is consistent with previous findings that more frequent and stronger Asian export events occurred in spring than other seasons^9^,^30^,^31^. Such rapid atmospheric movements in spring also bring BC aerosols from Asian source regions to the Pacific basin, and each spring maximum is consisted of multiple sBC* plumes with each lasting 3–7 days.

The inter-annual variability shown is larger over high- or low-latitude areas than mid-latitudes. For instance, in the year of 2008, there are extraordinarily high levels of sBC* plumes over high latitudes during spring, whereas in the years of 2007, 2010 and 2012, strong biomass burning plumes from Southeastern Asia are observed. During summers, enhanced sBC* plumes were observed in 2012, while during winters, trans-Pacific transport of Asian plume is much stronger in 2008 than other years.

### Comparison to other black carbon databases

**Figure 3:**
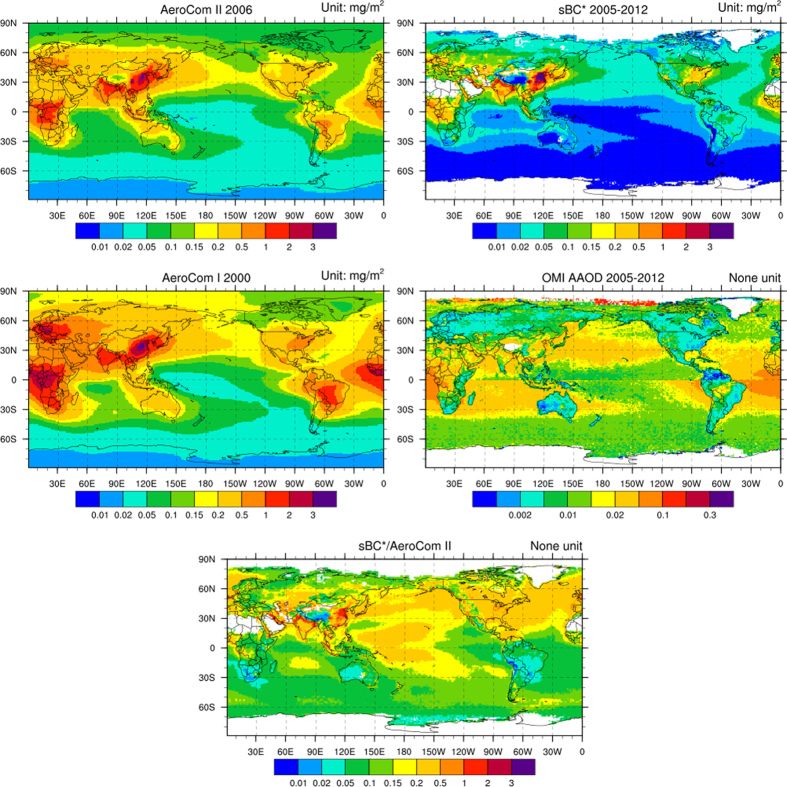
Global distribution of AeroCom II column BC (unit: mg·m^−2^), column sBC*(unit: mg·m^−2^), AeroCom I column BC (unit: mg·m^−2^), OMI AAOD averaged from 2005 to 2012 and the ratio between column sBC* and AeroCom II column BC (The NCAR Command Language)^40^.

So far, there is no direct global BC concentration dataset available but there are several sets of indirect global data, including Aerosol Comparisons between Observations and Models (AeroCom) Phase I/II and OMI Absorption Aerosol Optical Depth (AAOD). AeroCom provides median global BC concentration merged from a series of chemical models and the Phase I and II results are based on meteorological field and emission inventory in 2000 and 2006 respectively^4^,^15^,^32^,^33^. Although OMI AAOD products are poorly validated, especially over the ocean where there are much more missing points than over land areas, they offer valuable global coverage information on absorbing aerosols. In Fig. 3, global average map of sBC* is compared with AeroCom BC Phase I, II and OMI AAOD. Over Northern Pacific, AeroCom and sBC* (both significantly different from AAOD) have similar pattern of Asian exports of BC to the mid-Pacific. The pattern over the southern Pacific and other oceans and most continents also agrees though the data pool deriving sBC* only covers North Pacific. However, large scale discrepancies exist between harmonized model results and sBC* wherein the ratios between AeroCom BC and sBC* are about 2 to 5 over most oceanic and remote continental regions, but are reduced to 1–2 over the northern India and 0.5–1 over the eastern China. There is also a noticeable decrease in BC burden between two AeroCom phases. The discrepancies between sBC* and AeroCom, although less obvious when comparing with Aerocom Phase II, indicate potential overestimation of BC concentrations by models over remote Pacific. For other oceans, such as Atlantic and Indian Ocean, the bias is even larger and more direct observations are needed to validate such bias. On the other hand, OMI AAOD shows poor quality comparing to sBC* and Aerocom particularly over oceans, suggesting that it is affected by other absorbing aerosols and may not be an eligible tracer for black carbon aerosols over oceans.

### Inter-annual variability of sBC* over North Pacific

**Figure 4:**
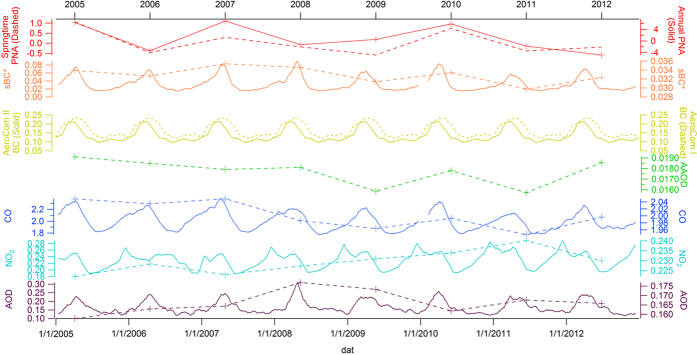
Temporal pattern of springtime/annual PNA (None unit), sBC* (mg/m^2^), AOD (None unit), tropospheric NO_2_ (x10^15^molecules/cm^2^), CO (x10^18^molecules/cm^2^), AAOD (None unit) over Northern Pacific (140°E–40°W, 0°N–60°N) from 2005 to 2012. 30-day running average is in solid lines and annual trend is in dashed line except for PNA.

The Pacific/ North American teleconnection pattern (PNA) is a mode of low-frequency variability in the Northern Hemisphere extratropics. While the positive phase indicates an enhanced East Asian jet stream and an eastward shift in the jet exit region toward the western United States, the negative phase indicates a westward retraction of that jet stream toward eastern Asia, blocking activity over the high latitudes of North Pacific, and a strong split-flow configuration over the central North Pacific. Therefore, PNA is positively associated with the strength and frequency of trans-Pacific transport events. Since Asian export is strongest in spring, we find the inter-annual variability of springtime PNA (averaged from March to May) is well correlated with the springtime sBC* (Fig. 4), indicating the strength of PNA affect the variability of black carbon forcing over the Pacific Ocean.

In Fig. 4, 30-day running average of sBC* is also compared with AOD, tropospheric NO_2_, CO, AAOD over Northern Pacific (140°E–160°W, 0°N–60°N). Daily AAOD is excluded here because there are very few daily data points over Pacific and their daily average is considered unreliable. Both sBC* and AeroCom BC peak in spring. Among AOD, CO and tropospheric NO_2_, annual trend of sBC* correlates best with CO. Meanwhile, the annual trend of CO agrees pretty well with springtime PNA, which suggests that East Asia jet stream in spring is the dominant factor influencing the inter-annual variability of CO over North Pacific and it could also influence exports of black carbon aerosols through similar mechanisms. Therefore, CO in Equation (1) serves an indicator of the strength of Asian outflow in considering both emission variability and meteorological conditions. However, when CO concentrations are sufficiently large, Equation (1) indicates that the variability of BC burden over the Pacific is largely controlled by removal processes. Sensitivity test on individual parameters in Equation (1) reveals that sBC* increases exponentially when AOD increases, which means high AOD over North Pacific is a good indicator of co-existed BC plumes. In Fig. 4, the spring peaks of AOD also vary consistently with the fluctuation of sBC* peak, especially in 2008, 2009 and 2011. This is because wet deposition of hydrophilic aerosols determines the distance that aged BC can travel. The temporal pattern of tropospheric NO_2_ appears to be much different and strong export of NO_2_ from Asia to Northern Pacific always happen in winter and spring. Massive coal consumption for heating and inactive photochemical reaction in Asia’s developing countries could explain the winter peak of NO_2_ and this could also indicate considerable amount of co-emitted BC aerosols with a slower aging rate.

The time-series of annual average sBC* reveals an approximately 10% variability over the eight years. Although HIPPO-3 suggests high loadings in 2010 than the rest two years, our findings shows a persistent BC loading over the northern Pacific and the corresponding BC forcing also remains stable during 2000s. It is also worth noting that there is a significant decreasing trend of CO over North Pacific, while the trend of sBC* is insignificant over the eight years.

## Discussion

The consistency between measured BC concentration by aircraft campaigns and co-located satellite retrievals confirms that high CO, AOD and tropospheric NO_2_ observed by satellites are associated with large co-existed BC plumes over the Pacific. Although the interaction among these air pollutants are extremely complicated and are subject to emission characteristics, meteorological conditions and numerous chemical reactions, the empirical equation (1) helps us find an “average” result of influences from all these factors on BC aerosols over North Pacific and the sBC* dataset reconstructed based on such an empirical approach provides us with unique information on the spatiotemporal distribution of black carbon globally. However, there are certain constraints on such application. Since Equation (1) is validated by HIPPO and A-FORCE measurements, there is a higher confidence in describing the spatial distribution of sBC* over the Pacific than other regions. The temporal characteristics are believed to be captured in the model since the original data spans over all seasons. It is also worth noting that satellite data sources chosen here mainly reflect the atmospheric composition in the mid- and upper-troposphere, where air pollutants mostly stay over oceanic areas. Over land, however, large percentage of the pollution is capped under the boundary layer. Therefore, sBC* is more reliable over remote oceanic places than land areas and it serves an unprecedented data source to constrain BC forcing over Pacific Ocean.

Comparison between sBC* and AeroCom model results reveals a 2–5 times overestimation over North Pacific and other oceanic regions by current BC models. Similar overestimation between HIPPO and AeroCom model over Pacific has also been reported by Want *et al*., 2014. Such a general overestimation informs us that BC climate forcing might be weaker than previous reports by present global models. Two direct radiative forcing (DRF) calculation of BC forcing over North Pacific (140°E–40°W, 0°N–60°N, 10.4% of total surface area) were conducted and the average BC DRF over North Pacific is 0.26 W/m^2^ based on AeroCom II BC input and 0.12 W/m^2^ based on sBC* input. Our result for AeroCom BC DRF is consistent with those by Wang *et al*.^6^, (Figure 11) and Bond *et al*.^2^, (Figure 17). However, the BC DRF derived from our sBC* dataset combining aircraft measurements and satellite retrievals is significantly lower than any previous results over North Pacific. And if such discrepancies are caused by some systematic model biases in BC emissions, evolution, or transport, similar overestimation could also be present in other remote oceans including Atlantic Ocean and Indian Ocean where the overall contribution is not negligible.

The comprehensive sBC* dataset also reveals a consistency among satellite observations, sBC* and climate index PNA, which suggests that the strength and frequency of the East Asia jet stream in spring is a potential factor dominating the annual average of Asian outflows over the Pacific, including BC plumes and other gaseous pollutants. In addition, the largest deviation of sBC* in the selected eight years is approximately 10% of the average while the seasonal variation could be as large as one magnitude of order. This illustrates a large, persistent but highly season-dependent BC forcing over the Pacific Ocean.

## Method

### Aircraft Campaigns

#### HIPPO

From 2009 to 2011, five independent HIAPER Pole-to-Pole Observation aircraft campaigns sampled the atmosphere over Pacific, from the North Pole to the coastal waters of Antarctica. Single-particle soot photometer (SP2) was used to measure BC mass mixing ratio. Respectively, five transects were conducted in January and November (2009), March/April (2010), June and August/September (2011). Over 700 vertical profiles were measured from the surface to 14 km every 2.2° latitude and every profile averagely consists of 200 BC measurements^34^. Here, we extract 341 BC profiles and calculate accumulative column BC concentration from the lowest altitude to the highest altitude of every profile by the unit of mg/m^2^. The latitude and longitude of such column BC concentration is defined by the mean of all measurements within the profile with a resolution by 1° × 1°. Therefore, the extracted column BC concentration matches well with satellite observations.

#### A-FORCE

From March 18 to April 25, 2009, the Aerosol Radiative Forcing in East Asia (A-FORCE) aircraft campaign sampled the atmosphere over Yellow Sea, the East China Sea and the western Pacific Ocean. Size distribution of BC particles was measured using SP2 and total BC mass concentration was derived by assuming a BC density of 2.0 g cm^−3^ 35. 34 vertical profiles over Asian outflow region have been extracted using similar method. The A-FORCE data can be used to validate BC column concentrations over Western Pacific.

#### Column Integration

The original data from aircraft campaigns consist of a series of BC mass mixing ratio measurement along with longitude, latitude, altitude and ambient pressure. The flight trajectory generally follows a saw-shaped pattern ascending from above the sea surface to the upper troposphere and then descending downward to lower troposphere and such a pattern repeats as the aircraft travel through the ocean. We treat a consecutive and complete set of measurements going from the lower troposphere to the upper troposphere (or the other way around) as a column and the column concentration is defined by summing up the BC aerosol loadings in the column. The geological location of the column is defined by the measurements with a resolution of 1° × 1°.

### Satellite Observations

#### Carbon Monoxide

There have been several global CO datasets from satellites, including the Measurements Of Pollution In The Troposphere (MOPITT), the Atmospheric Infrared Sounder (AIRS), the Tropospheric Emission Spectrometer (TES) and the SCanning Imaging Absorption SpectroMeter for Atmospheric CHartographY (SCIAMACHY). Here we use AIRS dataset for CO satellite observations and the reasons are: (1) AIRS has the longest cross-track scanning swath and a cloud clearing capability achieves 70% effective daily coverage, which ensures the least missing points in a daily map, AIRS version5 algorithms have been much improved and validation only shows a biased about 6–10% between 900 hPa and 300 hPa with a root-mean-square error of 8–12%^25^,^36^. Daily coverage and reliable validation are also important criteria when choosing satellite data sources for other air pollutants. Here, we use daily AIRS CO dataset (AIRSX3STD.005) downloaded from ftp://acdisc.gsfc.nasa.gov/ftp/data/s4pa/Aqua_AIRS_Level3/AIRX3STD.005/. The geological resolution is 1° × 1°.

#### Aerosol Optical Depth

The Moderate Resolution Imaging Spectroradiometer (MODIS) is a sensor with the ability to characterize the spatial/temporal characteristics of the global aerosol field and has been improving our understanding of global aerosol optical depth. Validation shows remarkably good agreement with ground-based AERONET, especially over Ocean^25^. MODIS Level 3 Daily products are provided at 1° × 1° resolution and are compatible to other data sources here. However, it takes 16 days to finish a global coverage for MODIS and it results in too many missing points in daily maps, which will largely damage our data pool for analysis. Therefore, we synergize two L3 MODIS AOD datasets from Aqua (MYD08_D3) and Terra (MOD08_D3) together by their average value. Data are downloaded from ftp://ladsweb.nascom.nasa.gov/allData/51/MOD08_D3 and ftp://ladsweb.nascom.nasa.gov/allData/51/MYD08_D3, respectively.

#### Tropospheric Nitrogen Dioxide

Ozone Monitoring Instrument (OMI) onboard the Earth Observing System (EOS) Aura satellite provides valuable information on tropospheric NO_2_. Direct and indirect validation researches found good correlation and no obvious bias between OMI tropospheric NO_2_ and *in-situ* measurements^26^,^37^,^38^.

#### Statistical Analysis

Nonlinear multivariate analysis is conducted to find the best fit of parameters between satellite observations and BC measurements. BC column mass concentration is transformed into its natural logarithm value and several models have been tested including exponential terms and quadratic terms. Equation (1) is the equivalent exponential form of the original natural logarithm equation: 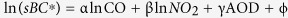. Based on the empirical model, global satellite retrievals on CO, AOD and tropospheric NO_2_ from 2005 to 2012 are applied to infer a historical daily BC dataset. Because of a high reflective surface in polar areas, there are many missing values of AOD there, especially during wintertime. In addition, AIRS CO observations are missing from 01/10/2010 to 01/25/2010. Column BC mass concentration is defined as missing value if any satellite observation is unavailable.

#### BC Direct Radiative Forcing

We use the offline Rapid Radiative Transfer Model for GCMs (RRTMG)^39^ to perform direct radiative forcing (DRF) calculations with a resolution of 1.9° × 2.5°. The calculation is based on perturbations of radiative fluxes by BC at the top-of-atmosphere (TOA) comparing with a zero-BC base case. There are two different sets of BC fields as input: one is the original mass mixing ratio of BC from harmonized median AeroCom II, and the other is an AeroCom II vertical profile but scaled by sBC* column concentration. The average DRF over North Pacific is obtained by weighting the total BC DRF over North Pacific by the corresponding area.

## Additional Information

**How to cite this article:** Li, Z. *et al*. A potential large and persistent black carbon forcing over Northern Pacific inferred from satellite observations. *Sci. Rep.* **7**, 43429; doi: 10.1038/srep43429 (2017).

**Publisher's note:** Springer Nature remains neutral with regard to jurisdictional claims in published maps and institutional affiliations.

## References

[b1] Ramanathan V. & Carmichael G. Global and regional climate changes due to black carbon. Nature geoscience 1, 221–227 (2008).

[b2] Bond T. C. . Bounding the role of black carbon in the climate system: A scientific assessment. Journal of Geophysical Research: Atmospheres 118, 5380–5552 (2013).

[b3] Schulz M. . Radiative forcing by aerosols as derived from the AeroCom present-day and pre-industrial simulations. Atmospheric Chemistry and Physics 6, 5225–5246 (2006).

[b4] Myhre G. . Radiative forcing of the direct aerosol effect from AeroCom Phase II simulations. Atmospheric Chemistry and Physics 13, 1853 (2013).

[b5] He C. . Black carbon radiative forcing over the Tibetan Plateau. Geophysical Research Letters 41, 7806–7813 (2014).

[b6] Wang Q. . Global budget and radiative forcing of black carbon aerosol: Constraints from pole‐to‐pole (HIPPO) observations across the Pacific. Journal of Geophysical Research: Atmospheres 119, 195–206 (2014).

[b7] He C., Li Q., Liou K., Qi L., Tao S. & Schwarz J. Microphysics-based black carbon aging in a global CTM: constraints from HIPPO observations and implications for global black carbon budget. Atmospheric Chemistry & Physics Discussions 15 (2015).

[b8] He C. . Variation of the radiative properties during black carbon aging: theoretical and experimental intercomparison. Atmospheric Chemistry and Physics 15, 11967–11980 (2015).

[b9] Liu H., Jacob D. J., Bey I., Yantosca R. M., Duncan B. N. & Sachse G. W. Transport pathways for Asian pollution outflow over the Pacific: Interannual and seasonal variations. Journal of Geophysical Research: Atmospheres 108 (2003).

[b10] Wang R. . Black carbon emissions in China from 1949 to 2050. Environmental science & technology 46, 7595–7603 (2012). 22730898 10.1021/es3003684

[b11] Ding A. J. . Enhanced haze pollution by black carbon in megacities in China. Geophys. Res. Lett. 43, 2873–2879 (2016).

[b12] Yu S., Saxena V. K. & Zhao Z. A comparison of signals of regional aerosol‐induced forcing in eastern China and the southeastern United States. Geophysical Research Letters. 28(4), 713–716 (2001).

[b13] Liang Q., Jaeglé L. & Wallace J. M. Meteorological indices for Asian outflow and transpacific transport on daily to interannual timescales. Journal of Geophysical Research: Atmospheres 110 (2005).

[b14] Wuebbles D. J., Lei H. & Lin J. Intercontinental transport of aerosols and photochemical oxidants from Asia and its consequences. Environmental pollution 150, 65–84 (2007). 17714840 10.1016/j.envpol.2007.06.066

[b15] Schwarz J. . Global-scale seasonally resolved black carbon vertical profiles over the Pacific. Geophysical research letters 40, 5542–5547 (2013). 26311916 10.1002/2013GL057775 PMC4542199

[b16] Remer L. A. . The MODIS aerosol algorithm, products, and validation. Journal of the atmospheric sciences 62, 947–973 (2005).

[b17] Koch D. . Evaluation of black carbon estimations in global aerosol models. Atmospheric Chemistry and Physics 9, 9001–9026 (2009).

[b18] Jethva H., Torres O. & Ahn C. Global assessment of OMI aerosol single‐scattering albedo using ground-based AERONET inversion. Journal of Geophysical Research: Atmospheres 119, 9020–9040 (2014).

[b19] Latha K. M. & Badarinath K. Correlation between black carbon aerosols, carbon monoxide and tropospheric ozone over a tropical urban site. Atmospheric research 71, 265–274 (2004).

[b20] Hadley O. . Trans-Pacific transport of black carbon and fine aerosols (D < 2.5 μm) into North America. Journal of Geophysical Research: Atmospheres 112 (2007).

[b21] Spackman J. . Empirical correlations between black carbon aerosol and carbon monoxide in the lower and middle troposphere. Geophysical Research Letters 35 (2008).

[b22] Verma R. . Seasonal variations of the transport of black carbon and carbon monoxide from the Asian continent to the western Pacific in the boundary layer. Journal of Geophysical Research: Atmospheres 116 (2011).

[b23] Pan X. . Correlation of black carbon aerosol and carbon monoxide in the high-altitude environment of Mt. Huang in Eastern China. Atmospheric Chemistry and Physics 11, 9735–9747 (2011).

[b24] Clarisse L. . Intercontinental transport of anthropogenic sulfur dioxide and other pollutants: An infrared remote sensing case study. Geophysical research letters 38 (2011).

[b25] McMillan W. . Daily global maps of carbon monoxide from NASA’s Atmospheric Infrared Sounder. Geophysical Research Letters 32 (2005).

[b26] Boersma K. . Near-real time retrieval of tropospheric NO 2 from OMI. Atmospheric Chemistry and Physics Discussions 6, 12301–12345 (2006).

[b27] Liu Y., Liu J. & Tao S. Interannual variability of summertime aerosol optical depth over East Asia during 2000–2011: a potential influence from El Niño Southern Oscillation. Environmental Research Letters 8, 044034 (2013).

[b28] Shen Z. . Analysis of transpacific transport of black carbon during HIPPO-3: implications for black carbon aging. Atmospheric Chemistry and Physics 14, 6315–6327 (2014).

[b29] Soja A. J. . Estimating fire emissions and disparities in boreal Siberia (1998–2002). Journal of Geophysical Research: Atmospheres 109 (2004).

[b30] Bey I. . Asian chemical outflow to the Pacific in spring: Origins, pathways, and budgets. Journal of Geophysical Research: Atmospheres 106(D19), 23–097 (2001).

[b31] Liu J., Mauzerall D. L. & Horowitz L. W. Analysis of seasonal and interannual variability in transpacific transport. Journal of Geophysical Research: Atmospheres 110 (2005).

[b32] Dentener F. . Emissions of primary aerosol and precursor gases in the years 2000 and 1750 prescribed data-sets for AeroCom. Atmospheric Chemistry and Physics 6, 4321–4344 (2006).

[b33] Textor C. . The effect of harmonized emissions on aerosol properties in global models–an AeroCom experiment. Atmospheric Chemistry and Physics 7, 4489–4501 (2007).

[b34] Wofsy S. C. HIAPER Pole-to-Pole Observations (HIPPO): fine-grained, global-scale measurements of climatically important atmospheric gases and aerosols. Philosophical Transactions of the Royal Society of London A: Mathematical, Physical and Engineering Sciences 369, 2073–2086 (2011). 10.1098/rsta.2010.0313 21502177

[b35] Oshima N. . Wet removal of black carbon in Asian outflow: Aerosol Radiative Forcing in East Asia (A-FORCE) aircraft campaign. Journal of Geophysical Research: Atmospheres 117 (2012).

[b36] McMillan W. W., Evans K. D., Barnet C. D., Maddy E. S., Sachse G. W. & Diskin G. S. Validating the AIRS Version 5 CO retrieval with DACOM *in situ* measurements during INTEX-A and-B. IEEE Transactions on Geoscience and Remote Sensing 49, 2802–2813 (2011).

[b37] Lamsal L. . Ground-level nitrogen dioxide concentrations inferred from the satellite-borne Ozone Monitoring Instrument. Journal of Geophysical Research: Atmospheres 113 (2008). 10.1002/2014JD022913 PMC5034499 27708989

[b38] Lamsal L. . Indirect validation of tropospheric nitrogen dioxide retrieved from the OMI satellite instrument: Insight into the seasonal variation of nitrogen oxides at northern midlatitudes. Journal of Geophysical Research: Atmospheres 115 (2010).

[b39] Iacono M. J., Delamere J. S., Mlawer E. J., Shephard M. W., Clough S. A. & Collins W. D. Radiative forcing by long-lived greenhouse gases: Calculations with the AER radiative transfer models. Journal of Geophysical Research: Atmospheres 113 (2008).

[b40] The NCAR Command Language (Version 6.3.0) [Software]. Boulder, Colorado: UCAR/NCAR/CISL/TDD. http://dx.doi.org/10.5065/D6WD3XH5 (2016)

